# Toxicological Evaluation of Acetylsalicylic Acid in Non-Target Organisms: Chronic Exposure on *Mytilus galloprovincialis* (Lamarck, 1819)

**DOI:** 10.3389/fphys.2022.920952

**Published:** 2022-07-11

**Authors:** M. Pagano, S. Savoca, F. Impellitteri, M. Albano, G. Capillo, C. Faggio

**Affiliations:** ^1^ Department of Chemical, Biological, Pharmaceutical and Environmental Sciences, University of Messina, Messina, Italy; ^2^ Department of Biomedical, Dental and Morphological and Functional Imaging, University of Messina, Messina, Italy; ^3^ Institute for Marine Biological Resources and Biotechnology (IRBIM), National Research Council (CNR), Messina, Italy; ^4^ Department of Veterinary Sciences, Polo Universitario Dell’Annunziata, University of Messina, Messina, Italy

**Keywords:** mediterranean mussel, drugs, histology, regulation volume decrease, viability analyses

## Abstract

Pharmaceuticals are now considered to be established contaminants, and their presence in water poses a real risk not only to the marine ecosystem, as they may adversely affect non-target organisms that are exposed to them, but also indirectly to humans. This is particularly true for the model organism considered in this work, *Mytilus galloprovincialis* (Lamarck, 1819), a suspensivore and bioaccumulating organism that enters the human food chain. Among the most commonly used over-the-counter medicines, anti-inflammatory drugs certainly feature prominently, with acetylsalicylic acid (ASA) at the top. In this work, *M. galloprovincialis* specimens were exposed to two concentrations of ASA (10 and 100 μg/L) for 10 and 20 days to evaluate possible alterations in the decrease in regulatory volume (RVD) in digestive gland cells and cell viability of both these cells and hemocytes. In addition, the histopathological condition index of the gills and digestive gland was evaluated. The data obtained showed that chronic exposure to ASA did not alter the cell viability of hemocytes and digestive gland cells but alters the physiological mechanisms of volume regulation in the digestive gland and, in addition, a time-dose reaction to ASA in the gills and digestive gland showing numerous alterations such as lipofuscin deposits and hemocyte infiltration was found. These results confirm the potential toxicity to the marine biota, highlighting the necessity to deepen the knowledge regarding the link between over-the-counter pharmaceuticals and non-target organisms.

## 1 Introduction

The contamination of wastewater and drinking water is a major environmental and public health problem. The main cause of contamination is anthropogenic. The various contaminants present in urban wastewater are called contaminants of emerging concern (CECs) and originate from industrial, hospital, and domestic wastewater, which end up in the marine and terrestrial environment (Rock et al., 2009). However, most of the chemicals released into the environment are not monitored, and their diffusion, interaction, and effects on ecosystems are poorly explored (Carere et al., 2019).

CECs include different classes of chemicals such as drugs, nanomaterials, microplastics, pesticides, flame retardants, perfluorinated compounds, cosmetic products, and many others (Faggio et al., 2016; Pagano et al., 2016, Pagano et al., 2020; Stara et al., 2020; Savoca et al., 2021). Generally, the concentrations of these substances are not reduced in wastewater treatment plants (Capillo et al., 2014, 2018; Albano et al., 2021a; Spanò et al., 2021).

Among drugs, the most commonly found in the aquatic environment are anti-inflammatories (e.g., diclofenac or ibuprofen), antibiotics (e.g., erythromycin, azithromycin), beta-blockers (metoprolol), lipid regulators (gemfibrozil), antidepressants (fluoxetine), antiepileptics (carbamazepine), diuretics, antidiabetics, synthetic hormones (e.g., alpha estradiol), and others (Aliko et al., 2021). Some of these active pharmaceutical ingredients may also have bioaccumulative properties and therefore potentially have the ability to enter the aquatic or terrestrial food chains (Klimaszyk and Rzymski, 2017; Carere et al., 2019) and through biomagnification phenomena may become dangerous to animal and human health (Zenker et al., 2014; XueLi and Hong, 2016; Aliko et al., 2021).

Mussels have also been reported as suitable test organisms for use in ecotoxicological assays due to their wide distribution, resistance to many contaminants, ease in capturing and maintenance under laboratory conditions, and being useful for characterizing the full ecotoxicological potential of drugs.

Acetylsalicylic acid (ASA) is a non-steroidal anti-inflammatory drug among the most widely produced and consumed drugs, in the range of several kilotons per year (Cleuvers, 2004).

ASA is an anti-inflammatory drug that permanently inactivates COX-2 through acetylation of a serine located near the catalytic site of the enzyme. Therefore, the duration of the inhibitory effect of aspirin depends on the rate of *de novo* synthesis of the enzyme by the target cells after the drug’s rapid disappearance from circulation (FitzGerald and Patrono, 2001; Patrignani and Patrono, 2015).

ASA, like other active pharmaceutical ingredients, has also been found in surface and groundwater, including sources of drinking water (Klimaszyk and Rzymski, 2017). The main source of ASA pollution is from industrial, urban, and agricultural spills, but it has also been found in municipal, livestock, and pharmaceutical and hospital wastewater treatment plants, as reported by the free database of the German Environment Agency, available from: https://www.umweltbundesamt.de/dokument/database-pharmaceuticals-in-the-environment-excel. In European waters, the estimated ASA concentration is 80.4 μg/L (Stuer-Lauridsen et al., 2000).

*M. galloprovincialis* has been poorly studied in the possible interaction with ASA. Piedade et al. (2020) show that acute exposures do not alter the animal’s oxidative metabolism. In contrast, exposures of *M. galloprovincialis* to salicylic acid not only reduce respiration capacity but also the normal antioxidant balance (Freitas et al., 2020b, 2019).

Since CECs can persist in the aquatic environment for long periods, the health concern about ASA and ASA-like contaminants is due to their implications for non-target aquatic organisms, that is, organisms that are not intended to be affected by these xenobiotics. Due to their feeding mode, filter-feeder organisms could be particularly exposed and sensitive to this class of pollutants (Deeds et al., 2008; Albano, et al., 2021b; Sauvey et al., 2021). For this reason, the Mediterranean mussel (*Mytilus galloprovincialis*, Lamarck, 1819) has been chosen as a model organism in this study. *Mytilus galloprovincialis* is characterized by physiological and cellular mechanisms that can be used as markers to evaluate the possible effects of pollutants (Freitas et al., 2021, 2020b, 2020a, 2019; Pagano et al., 2020, 2017).

The present study aims to assess the effect of two different concentrations of ASA, one less than estimated ASA1: 10 μg/L and the other greater than estimated ASA2: 100 μg/L after chronic exposure (10 and 20 days) on the fitness of *M. galloprovincialis* to provide the basic knowledge about non-target organisms and ecosystem responses to this contaminant.

## 2 Materials and Methods

### 2.1 Experimental Design

*Mytilus galloprovincialis* specimens, 5.60 ± 0.40 cm shell length, were obtained from the meromitic marine coastal lagoon named ‘‘Faro Lake” from a local bivalve mollusc farm (company Farau Srl, Frutti di Mare, Messina, Italy).

The Faro lagoon is an area exploited for bivalve rearing and cultivation (D’Iglio et al., 2022; Sanfilippo et al., 2022; Savoca et al., 2020).

One hundred-twenty specimens of mussels were maintained in 30 L aquaria filled with continuously aerated brackish water (salinity 32.96 ± 0.31 PSU) in the laboratory with daylight exposure 12 h light:12 h dark and temperature 18 ± 1°C for 7 days acclimation before the start of any experimental procedure.

After acclimation, 30 mussels were randomly selected and placed into each of the six aquaria (three experimental groups in duplicated) containing 20 L continuously aerated brackish water. The mussels were exposed to concentrations of ASA (minimum 99.5%) (Sigma-Aldrich, Darmstadt, Germany): control: 0 μg/L; ASA1: 10 μg/L; ASA2: 100 μg/L for 20 days. Thirteen mussel samples were sampled for laboratory analysis immediately before the transfer for the experimental exposure on 20 L aquaria (T0) after 10 (T1) and 20 (T2) days of exposure to ASA.

### 2.2 Hemolymph Collection

Hemolymph samples were collected from five mussels from each experimental group. Two pools for each experimental group were used for analyses. The hemolymph was collected from the anterior adductor muscle with a 23-gage needle to a 1-ml plastic syringe. Once collected, it was placed in tubes and immediately centrifuged at 1,000 rpm for 10 min. The pellet was resuspended in 1.5 ml of physiologic saline solution (NaCl 550 mM; KCl 12.5 mM; MgSO_4_ 8 mM; CaCl_2_ 4 mM; glucose 10 mM; HEPES 20 mM; and π= 1,100 mOsm).

### 2.3 Cell Viability Assays

The experiments used hemolymph and digestive gland cells of mussels. The viability of hemolymph and isolated digestive cells was evaluated by 1) the trypan blue (TB) exclusion method by microscopic observation and 2) the stability of the lysosomal membrane by neutral red (NR) retention assay by microscopic observation, according to Faggio et al. (2016).

### 2.4 Isolation of Digestive Cells and Regulation of Volume Decrease (RVD) Experiments

Digestive glands of four animals from each group were isolated according to the method of Torre et al. (2013), with slight modifications by Pagano et al. (2017). The cells were observed by using a light microscope (Carl Zeiss Axioskop 20, Wetzlar, Germany) connected to a Canon 550D camera that digitized the image to a PC. Individual cells were selected, and the images were taken at 0 and 3 s in isotonic solution; afterward, the solution was rapidly changed with a hypotonic solution (800 mOsm), and the image was taken every 1 min for the first 10 min after the change of the solution and after every 5 min for 20 min. The profiles of the cells were drawn with the aid of ImageJ (NIH, Bethesda, MD, United States). The data are reported as the relative area Aexp/Ai; indeed, the cell areas for each experimental condition (Aexp) were compared to the areas measured in isotonic solution (Ai) at the beginning of the experiment.

### 2.5 Histology

Immediately after hemolymph sampling, the gills and digestive glands were quickly removed from ice and stored and fixed in immunofix (paraformaldehyde 4% in phosphate-buffered saline, Bio-Optica, Milan, Italy) for 12 h at room temperature for histopathological condition evaluation. An investigation under histological conditions of digestive glands and gills was performed. Sampled fractions of both tissues from each treatment group were collected in triplicate from three specimens. Tissues were embedded in paraffin and successively sectioned to 5-μm sections by using a rotative microtome (Leica, RM2235). The obtained sections were stained using hematoxylin and eosin for a qualitative histopathological examination using a light microscope (Leitz Diaplan, Germany). For detailed procedures, see Pagano et al. (2016), Lauriano et al. (2019), Zaccone et al. (2015).

#### 2.5.1 Histopathological Condition Indices

For the evaluation of each individual histopathological index (Ih), a semi-quantitative weighted indices approach, initially described by Bernet et al. (1999) for fish and later modified by Costa et al. (2013), was applied. The Ih was calculated for both organs separately (gills and digestive gland) and related to “reaction patterns”: morphological epithelial modifications (gills) and tubule and intertubular tissue alterations (digestive gland). Through microscopic observation of the previously obtained sections, a weight (based on its biological importance) was assigned to each detected alteration, with a value ranging between 1 (minimum severity) and 3 (maximum severity) and a score (degree of dissemination) with values between 0 (alteration not detected) and 6 (alteration diffuse). The weights used have been based on observations collected in this experiment and partially on the literature about both invertebrate Costa et al. (2013) and vertebrate histopathology and are shown in Table1. Further details of the formula for the assessment of histopathological condition indices were reported by Stara et al. (2019).

**TABLE 1:** Reaction patterns. Weights assigned to each digestive gland and gill alteration.

Digestive gland	—	Gill	
Alteration	Weight	Alteration	Weight
Tubule alterations		Cellular and morphological changes	
Epithelial cell hyperplasia	2	Epithelial cell hyperplasia	2
Epithelial cell hypertrophy	2	Vacuolation	1
Brown cells	1	Hemocyte infiltration	1
Hemocyte infiltration	1	Granulocytoma	2
Lipofuscin aggregates	1	Lipofuscin aggregates	1
Necrosis	3	Fibrosis	2
Tubule regression	2	Necrosis	3
Intertubular tissue changes		Loss of epithelia	3
Hemocyte infiltration	1	Lamellar fusion	1
Lipofuscin aggregates	1	Lamellar deformation	1
Brown cells	1	Epithelial detachment	1
Fibrosis	2	—	
Necrosis	3	—	
Granulocytoma	2	—	

### 2.6 Statistical Analyses

The statistical analyses of results were performed using two-way ANOVA followed by the Bonferroni test for pairwise comparisons among experimental conditions in RVD assay and an unpaired *t*-test for comparisons in viability assays. Package Prism, Version 8.2.1 (GraphPad Software Ldt., La Jolla, CA 92037, United States) was used for statistical analysis. The data of histopathological indices (Ih) were analyzed using two-way ANOVA followed by Tukey’s *post hoc* test for multiple comparisons. *p*-value was set at *p* < 0.05. Statistical analysis was performed using the software package Prism, Version 8.2.1 (GraphPad Software Ldt., La Jolla, CA 92037, United States).

## 3 Results

### 3.1 Cell Viability Assays

As shown in Table 2, hemocytes maintain high viability values throughout the experiment at both drug concentrations. The same trend is evident in Table 3 for the cells of the digestive gland.

**TABLE 2:** Percentage of viability hemocytes in *Mytilus galloprovincialis* exposed to acetylsalicylic acid (control (0 mg/L); ASA1 (10 μg/L); and ASA2 (100 μg/L) by trypan blue (TB) and neutral red (NR) after 10 days (T1) and 20 days (T2) of exposure. One-way ANOVA was used to test the differences between control and treatment and the Tukey test. The values are presented as the mean ± SD (*n* = 5); significant differences compared with the control group value (*p* < 0.05).

Viability assays	Time of exposure	Test group		
		Control (0 mg/L)	ASA1 (10 μg/L)	ASA2 (100 μg/L)
TB	T1	100 ± 0	92.96 ± 1.00	99.00 ± 0.69
	T2	95.96 ± 0.49	95.92 ± 0.23	93.91 ± 0.13
NR	T1	100 ± 0	91.57 ± 0.75	99.00 ± 0.69
	T2	95.17 ± 0.11	97.79 ± 0.41	97.42 ± 0.11

**TABLE 3:** Percentage of viability of digestive cells in *Mytilus galloprovincialis* exposed to acetylsalicylic acid [Control (0 mg/L); ASA1 (10 μg/L); and ASA2 (100 μg/L)] by trypan blue (TB) and neutral red (NR) after 10 days (T1) and 20 days (T2) of exposure. The values are presented as the mean ± SE; significant differences compared with the control group value (*p* < 0.05).One-way ANOVA has been used to test the differences between control and treatment and the Tukey test.

Viability assays	Time of exposure	Test group		
		Control (0 mg/L)	ASA1 (10 μg/L)	ASA2 (100 μg/L)
TB	T1	100 ± 0	96.67 ± 0.50	97.89 ± 0.26
	T2	97.33 ± 0.21	98.86 ± 0.28	98.06 ± 0.18
NR	T1	100 ± 0	99.01 ± 0.62	98.33 ± 0.92
	T2	95.03 ± 0.13	98.28 ± 0.33	98.70 ± 0.03

### 3.2 RVD Experiment

**FIGURE 1:**
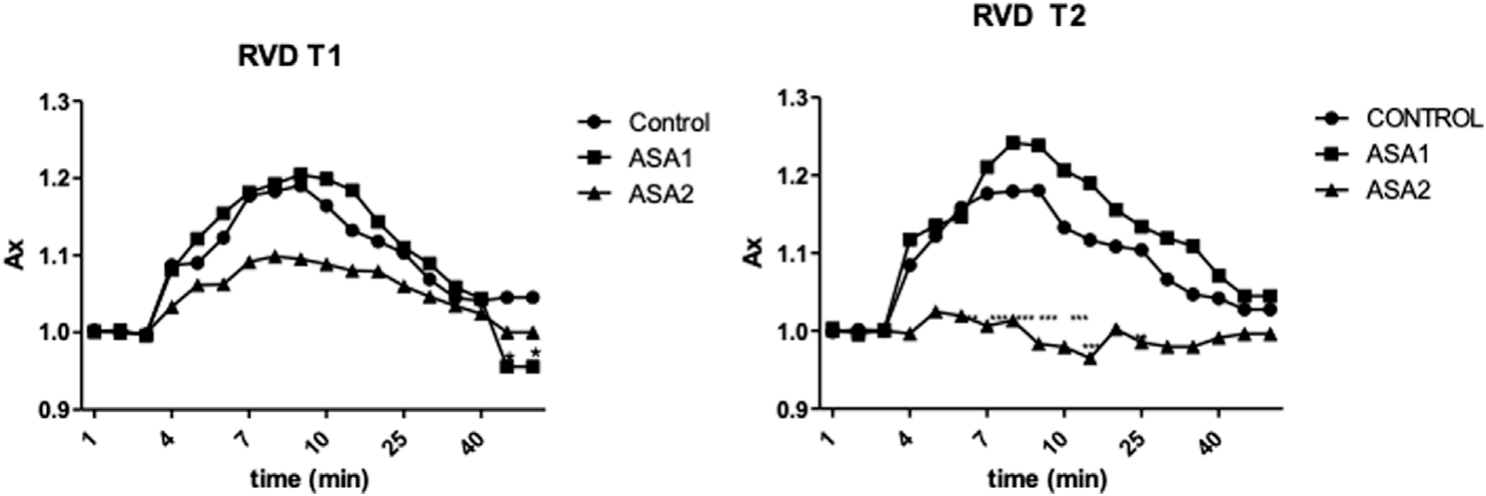
Relative changes in the area of digestive cells of *Mytilus galloprovincialis* exposed to a hypotonic solution for both exposure times, T1 and T2. The values are presented as the mean ± SD (*n* = 4); significant differences compared with the control group value (*p* < 0.05) (two-way ANOVA test). Control (0 μ*g* L^−1^) (◆), *ASA1.* (10 μ*g* L^−1^) (*▪*), and *ASA2* (100 μ*g* L^−1^) (▴)*.*

Digestive gland cells of Control and ASA1 organisms after exposure to hypotonic solution increased their volume by approximately 12% and then returned to their initial volume. This response was observable for both T1 and T2. On the other hand, the cells of the ASA2 group behaved differently at the two exposure times: at T1, the cells exposed to hypotonic solution swelled slowly to 10% of their volume and then returned to their initial conditions; at T2, the cells after washing with hypotonic solution were unable to swell (Figure 1).

### 3.3 Histology

**FIGURE 2:**
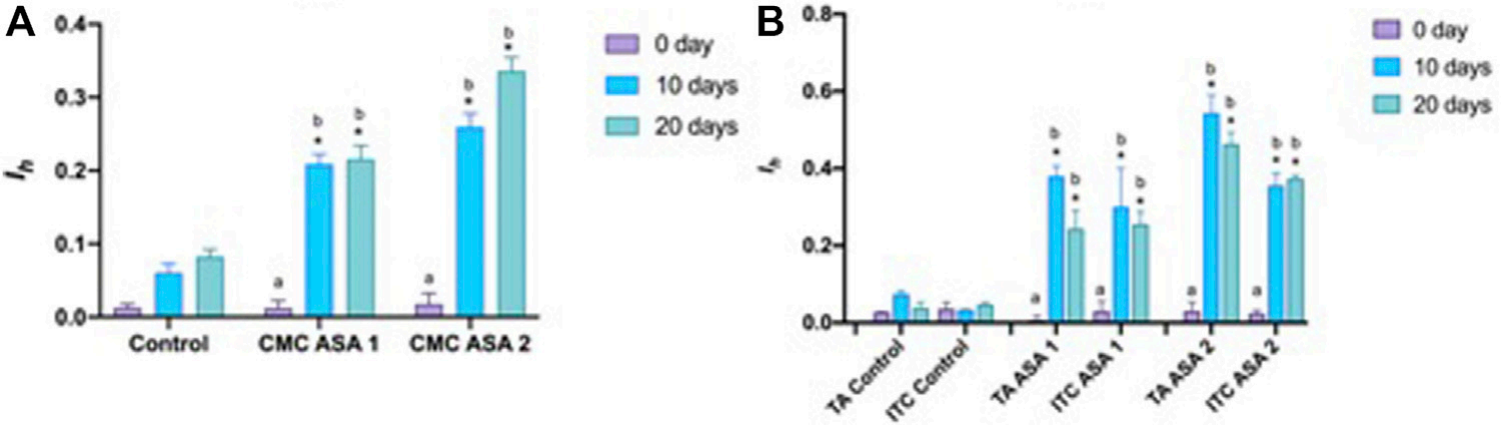
Histopathological condition index (I_h_) of tissues from *Mytilus galloprovincialis* exposed to acetylsalicylic acid (ASA): **(A)** gills and **(B)** digestive gland. The values are shown as mean ± SD (*n* = 3); stars represent significant differences between the treatment and control groups at the same time point. Letters are only present in the case of significant statistical differences. Different small letters indicate significant differences between time points within the same treatment group. Differences were considered significant when *p* < 0.05 (two-way ANOVA test/Tukey’s multiple comparisons test). ASA1 (ASA 10 μg L^−1^) and ASA2 (ASA 100 μg L^−1^). CMC, cellular and morphological changes; TA, tubular alteration; ITC, intertubular tissue changes.

**FIGURE 3:**
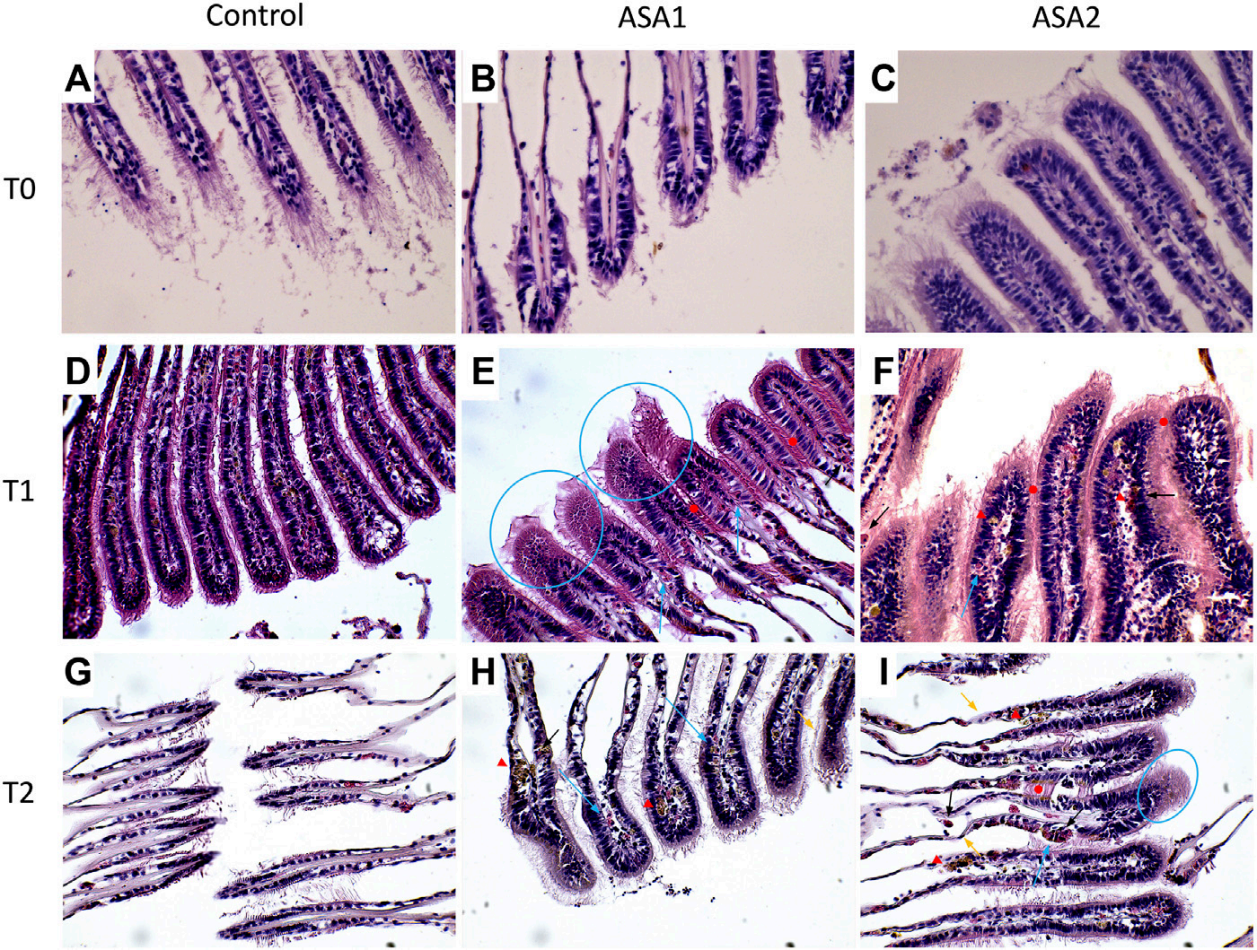
*Mytilus galloprovincialis* gill sections after ASA exposure. Representative images: **(A–C)** are, respectively, Control, ASA1, and ASA2, 0-day exposure; **(D–F)** are, respectively, Control, ASA1, and ASA2, 10-day exposure; **(G–I)** are, respectively, Control, ASA1, and ASA2, 20-day exposure. ASA1 (ASA 10 μg L^−1^) and ASA2 (ASA 100 μg L^−1^). Magnification ×40. Blue arrows highlight hemocyte infiltrations, blue circles highlight vacuolation, red arrowheads highlight lipofuscin aggregates, black arrows highlight granulocyte infiltrations, orange arrows highlight epithelial alterations, and red filled circles highlight lamellar fusion.

Histopathological alterations detected are shown in Figure 3. Histopathological condition index (I_h_) results are shown in Table 4. A time- and concentration-dependent reaction to ASA was detected in both the digestive gland and gills when compared to the control (*p* < 0.05) (Figure 2). I_h_ showed a trend dependent on different treatments and exposure times (*p* < 0.05) for both organs analyzed. In the gills, an increasing trend of I_h_ was observed, proportionally to increase in the exposure time and ASA concentration, although not statistically significant (Figure 3A). In the digestive gland tissue, no statistically significant differences were obtained comparing digestive tubule changes and intertubular tissue modifications in the group exposed to both concentrations tested, showing a marked decrease in I_h_ values at 20 days of exposure (Figure 3B).

**TABLE 4:** Histopathological condition index I_h_ of *Mytilus galloprovincialis* exposed to experimental concentrations of ASA. The values are presented as the means ± SD (*n* = 3). Significant differences compared with the control value set as *p* < 0.05 (*) are shown. ASA1 (ASA 10 μg L^−1^) and ASA2 (ASA 100 μg L^−1^).

Organ	Reaction pattern	Exposure time (days)	Test groups		
			Control	ASA1	ASA2
				(10 μg L^−1^)	(100 μg L^−1^)
Digestive gland	Tubule alterations	0	0.028 ± 0.0	0.07 ± 0.01*	0.03 ± 0.02*
		10	0.07 ± 0.0	0.38 ± 0.02*	0.54 ± 0.04*
		20	0.03 ± 0.01	0.24 ± 0.04*	0.46 ± 0.03*
	Intertubular tissue changes	0	0.03 ± 0.01	0.03 ± 0.02*	0.02 ± 0.0*
		10	0.03 ± 0.01	0.30 ± 0.10*	0.355 ± 0.03*
		20	0.04 ± 0.0	0.25 ± 0.03*	0.37 ± 0.0*
Gills	Cellular and morphological changes	0	0.013 ± 0.05	0.012 ± 0.01*	0.017 ± 0.015*
		10	0.06 ± 0.013	0.20 ± 0.01*	0.26 ± 0.01*
		20	0.082 ± 0.010	0.21 ± 0.01*	0.33 ± 0.019*

#### 3.3.1 Gills

Various serious alterations due to ASA exposure were recorded in gill tissues during the experiment. The most frequent histological modifications detected were alterations of epithelial structure, lamellar fusion, vacuolation, lipofuscin deposits, and hemocyte infiltration (Figure 3). An increasing trend of alterations was observed in I_h_ values, proportional to the increase in ASA concentration and exposure time, although no statistically significant differences were highlighted. Contrary to the digestive gland investigation, in the gills, no inversion on the increasing of the I_h_ trend was detected.

#### 3.3.2 Digestive Gland

**FIGURE 4:**
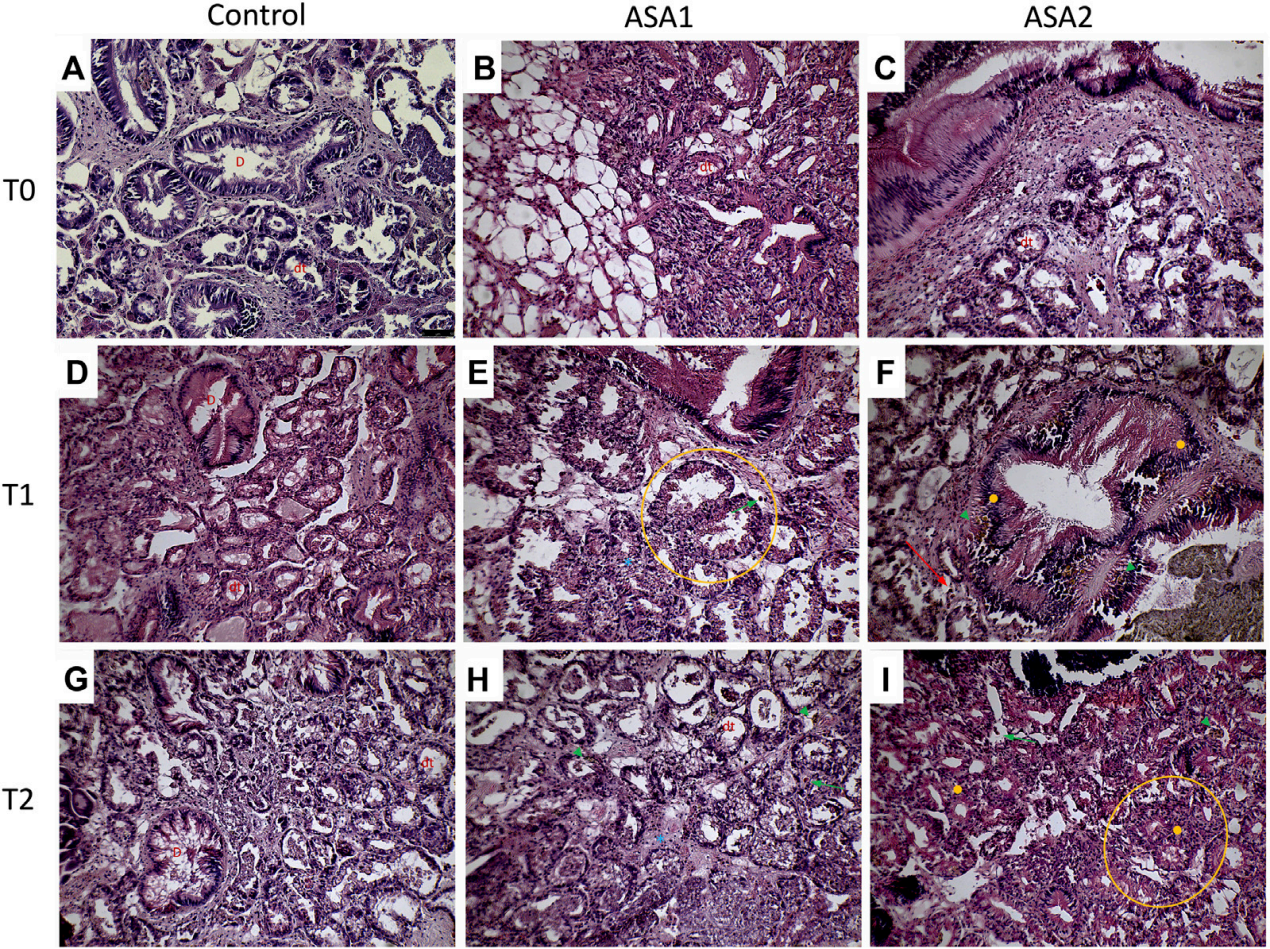
*Mytilus galloprovincialis* digestive gland sections from ASA exposure: representative figures. **(A–C)** are, respectively, Control, ASA1, and ASA2, 0-day exposure; **(D–F)** are, respectively, Control, ASA1, and ASA2, 10-day exposure; **(G–I)** are, respectively, Control, ASA 1, and ASA2, 20-day exposure. ASA 1 (ASA 10 μg L^−1^) and ASA2 (ASA 100 μg L^−1^). Magnification ×20. Orange circles highlight digestive tubule alteration, orange filled circles indicate hyperplasia, red arrows show brown cells, green arrowheads show lipofuscin aggregates, green arrows highlight hemocyte infiltrations, and blue asterisks highlight hypertrophy. D identifies duct; dt identifies digestive tubule.

Mainly present digestive gland alterations comprehended lipofuscin aggregates, hemocyte infiltration, and hyperplasia both in digestive tubule and intertubular tissues in exposed specimens. In the most severe cases, tubule regression, hypertrophy, and focal points of necrosis were observed in mussels exposed also to ASA1 (Figure 4). I_h_ in ASA1-exposed specimens was higher with respect to the ASA2 experiment, except for the sample ASA2 at 20 days of exposure. No significant discrepancies were obtained when comparing digestive tubule changes and intertubular tissue modifications.

## 4 Discussion

Acetylsalicylic acid is widely used by humans as an analgesic and is found in wastewater and urban effluents. Despite the larger amount of data on the occurrence of CECs in the aquatic environment, studies assessing their possible adverse effects on aquatic organisms are still poor and relatively limited. Few studies are available on the ASA effects on non-target organisms (Dittrich, 2018; Almeida et al., 2020; Piedade et al., 2020; Siddeswaran et al., 2020); instead, the effects of other anti-inflammatory agents have been studied (Freitas et al., 2019,2020b; Gonzalez-Rey and Bebianno, 2014; Mezzelani et al., 2018). Freitas et al. (2019), (2020a) showed that chronic exposure to salicylic acid (SA) reduces the respiration capacity of mussels and alters normal antioxidant balances and causes neurotoxic damage, and this will be amplified if temperatures are increased.

In our results, the cell viability assays highlighted the lack of interaction between the cells and the ASA. In fact, no significant alterations in cell viability were present at either concentration for any exposure time for both assays tested, and in contrast to other molluscs exposed to non-steroidal anti-inflammatory drugs, the lysosomal membranes were found to be intact (Parolini and Binelli, 2011; Parolini, 2020). Instead, there was a massive presence of hemocytes in the gills and in the digestive gland, demonstrated by histological examinations, which is the first indication of tissue inflammation (de Vico and Carella, 2012).

Bivalve gills are also involved in the alimentation process, filtering water and trapping particulate matter. In the bivalve mollusc’s anatomy, gills represent the first contact with mucosal surfaces by which the organism faces the surrounding water (Azevedo et al., 2015; Stara et al., 2020, 2019). This barrier can be crossed both by substances important for the organism and contaminants present in water (Canesi et al., 2012; Phuong et al., 2017; Azizi et al., 2018). Despite that gills are the primary site of contact with the pollutants, as reported by many authors, the main target of their accumulation and detoxification in bivalve molluscs is represented by the digestive gland (Faggio et al., 2016; Blanco et al., 2021; Stollberg et al., 2021). In addition, the digestive gland is also involved in the metabolism of heavy metals (Viarengo et al., 1981; Caricato et al., 2018). Histopathological modifications on these target tissues have been examined using the Ih as suggested by Costa et al. (2013). The I_h_ values determined for the histological alteration and reactions evaluated (Table 1) are shown in Table 4 and graphed in Figure 2. Regarding gills, I_h_ resulted higher, as expected, in ASA2 at 20 days exposure. In the digestive gland, I_h_ resulted higher in ASA2-treated specimens than ASA1, following a dose-dependent inflammation pattern. It is interesting to note how the I_h_ values detected for both examined structures of the digestive gland showed an unexpected trend. Indeed, the higher I_h_ values were, in both ASA1 and ASA2, the higher the exposure will be in the 10-day than in the 20- day experiment. It is also conceivable that for the tissue of the digestive gland, the 10 days exposed specimens suffer an acute reaction that was reduced in the 20-day experiment. It can be assumed that *M. galloprovincialis*, after an acute inflammatory response can tolerate exposure to ASA, as also reported in a previous study (Pagano et al., 2016; Bayne et al., 1979; Kumar Yadav, 2013.). The histological modifications reported in this study have been confirmed in *M. galloprovincialis* by our recent studies on the evaluation of the chronic exposure effect of some toxicants, both at acute and sub-lethal concentrations (Stara et al., 2020, 2021). Some other histological alterations were detected and related in this case to an inflammatory response to ASA.

Various stage inflammations have been detected in both organs examined (gills and digestive gland), related in our case to ASA exposure. These alterations could represent a first response to various pollutants and drugs in these organs, as already reported by other authors (Yasmeen, 2019; Abdel-Latif et al., 2020; Couch and Fournie, 2021). Regarding the gill tissue, these inflammations were mainly focal at low concentrations of toxicant characterized by vacuolation and sometimes widespread with infiltration of hemocytes and granulocytes in ASA2. The digestive glands showed a more diffused inflammation characterized by both hemocyte infiltration and hyperplasia, resulting in the loss of physiological anatomy. Nodular inflammations, such as granulocytomas, appear rarely and are not widespread compared to the results of other authors (Kumeiko et al., 2018; Yee-Duarte et al., 2018; Sendra et al., 2021). Considering that nodular inflammations occur from phagocytosis activity of hemocytes after a pathogen’s invasion (Rowley, 1996), that during their activity creates different sizes of aggregates in hemolymph and interstices (Galloway & Depledge, 2001), from the results, as expected, ASA exposure seems to not cause this aggregate formation. Despite this, some other authors have highlighted the aggregative properties of hemocytes under stimulation by acute or chronic exposure to xenobiotics (Auffret and Oubella, 1997; Carella et al., 2015). The brown cells were evident in digestive gland tissue, with higher frequency in intertubule spaces; these cells are highly present in the digestive gland of stressed organisms and are involved in recognition, accumulation, and detoxification of toxicants (Usheva and Frolova, 2006; de Vico and Carella, 2012). In addition, digestive gland cells exposed to hypotonic solutions can normally regulate their volume (Torre et al., 2013), but in the cells exposed to ASA2, there was an interaction response to the pollutant and the cellular mechanisms at T1, with the cells unable to regulate their volume. At T1, after hypostatic exposure, the cells swell less than in the other two conditions, and at T2, they cannot swell at all. *M. galloprovincialis*, being an osmoconforming organism, alterations in these capacities can be used as a parameter for assessing physiological changes (Pagano et al., 2016, 2017). It is as if long exposure to higher concentration of ASA has blocked the normal ionic efflux, also preventing the swelling of cells exposed to hypotonic concentration, behaving as an ion channel inhibitor as demonstrated by Torre et al. (2013).

Focusing on gill tissues, our analysis revealed an inflammatory status connected to ASA exposure. This reaction was characterized mainly by extended vacuolation, moderate deposits of lipofuscin, hemocyte infiltration, extended lamellar fusion, and modifications of epithelial morphology. Infiltrative inflammations characterized by various stages of hemocyte infiltrations are widely reported in gill tissues of molluscs exposed to environmental toxicants (Carnegie and Meyer, 2021; Khan et al., 2019; Kumeiko et al., 2018; Paviotti-Fischer et al., 2018). As for the digestive gland, the function of infiltrative hemocytes to phagocyte pathogens and/or foreign bodies is to initialize the organism’s response to xenobiotics, starting the multixenobiotic defense mechanism (MXDM) (Pain and Parant, 2003; Parant, 2022). The MXDM system represents a shield for cells and tissues from the adverse effect of toxicants through the reduction of their access and to favor their efflux (Pagano et al., 2016).

Destructive reactions at the expense of gill tissue morphology and functions, such as vacuolation, lamellar fusion, and loss of epithelial morphology, were already reported by several authors in bivalve molluscs, as common reactions to pollutant exposure (Khudhur et al., 2019; Joshy et al., 2022; Khan et al., 2018). The influence of ASA concentration on these modifications has followed a constantly increasing trend during our study. More interesting is their succession in relation to the exposure time. The massive presence of vacuolation found in our study after 10 days of exposure in higher presence suggests that this mechanism may be among the first inflammatory processes. On the contrary, lamellar fusion and modifications of the epithelial normal structure were found at the longest exposure time (20 days). This suggests that the highest functionally more severe modifications occur in the gill tissues of molluscs in a later stage of the inflammatory response process, as reported by other authors (Balamurugan and Subramanian, 2021; Pires et al., 2022).

In bivalve molluscs, lipofuscin formation is related mainly to cellular oxygen consumption (Katz et al., 1984), but several authors have studied how lipofuscin *in situ* also can represent a signal of primary reaction to the exposure, particularly to heavy metals or other pollutants (Mathew and Damodaran, 1997; Lomovasky et al., 2002; Husmann et al., 2012; Abdel-Latif et al., 2020). In this study, we found a steady increasing trend of the presence of lipofuscin aggregates in gills related both to ASA concentration and exposure time, which highlight its involvement in the generalized inflammatory response. In this case, the lipofuscin accumulation indicates a reaction to the oxidative damage caused by ASA exposure. Considering the lipofuscin more widely also as an age-related pigment linked to oxidation of by-products, further studies with prolonged exposure could also reveal its role in this process in molluscs.

## 5 Conclusion

The current study examined the chronic effect of acetylsalicylic acid on *M. galloprovincialis*. Our results show both physiological changes in the organism, such as altered regulation of cell volume and inflammation on a histological level, especially in the digestive gland. These results occur even at concentrations much lower than those estimated in the aquatic environment, reinforcing current assumptions about the need to investigate the effects of water contamination by drugs and/or their derived compounds. Therefore, the aim of this research was to increase knowledge of the ecotoxicological potential of one of the active pharmaceutical ingredients present in the water, acetylsalicylic acid, by studying physiology and possible histological alterations of *Mytilus galloprovincialis.*

## Data Availability

The raw data supporting the conclusion of this article will be made available by the authors, without undue reservation.
